# Colchicine and Atherosclerotic Coronary Artery Disease: An Updated Review

**DOI:** 10.3390/jcm14186396

**Published:** 2025-09-10

**Authors:** Simona Giubilato, Giuseppe Ciliberti, Pietro Scicchitano, Antonio Di Monaco, Federico Fortuni, Filippo Zilio, Claudio Mario Ciampi, Stefano Cangemi, Antonella Spinelli, Laura Gatto, Luca Franchin, Stefano Cornara, Michele Magnesa, Carlotta Sorini Dini, Enrica Vitale, Nicola Gasparetto, Giovanna Geraci, Roberta Rossini, Roberta Della Bona, Federico Nardi, Domenico Gabrielli, Michele Massimo Gulizia, Massimo Grimaldi, Fabrizio Oliva, Massimo Imazio

**Affiliations:** 1 Cardiology Department, Cannizzaro Hospital, 95126 Catania, Italy; 2 Clinica di Cardiologia e Aritmologia, AOU Ospedali Riuniti Umberto I-Lancisi-Salesi, 60126 Ancona, Italy; giuseppe.ciliberti@ospedaliriuniti.marche.it; 3 U.O.C. Cardiologia-UTIC P.O. “F. Perinei”, 70022 Altamura, Italy; piero.sc@hotmail.it; 4 Department of Cardiology, General Regional Hospital “F. Miulli”, Acquaviva delle Fonti, 70021 Bari, Italy; a.dimonaco@gmail.com (A.D.M.); m.grimaldi@miulli.it (M.G.); 5 Cardiology and Cardiovascular Pathophysiology, S. Maria della Misericordia Hospital, University of Perugia, 06129 Perugia, Italy; fortuni.ff9@gmail.com; 6 Department of Cardiology, Santa Chiara Hospital, APSS, 2, Largo Medaglie d’Oro, 38123 Trento, Italy; filippozi@yahoo.it; 7 Cardiology Department, Garibaldi Nesima Hospital, 95122 Catania, Italy; claudiomariociampi@gmail.com (C.M.C.); michele.gulizia60@gmail.com (M.M.G.); 8 Cardiology Unit, S. Antonio Abate Hospital, ASP Trapani, 91016 Erice, Italy; cangemi.stefano92@gmail.com (S.C.); giovannageraci@hotmail.com (G.G.); 9 Cardiology Department, San Filippo Neri Hospital, ASL Roma 1, 00135 Rome, Italy; antonellaspinelli81@gmail.com; 10 Cardiology Department, San Giovanni Addolorata Hospital, 00184 Rome, Italy; lauragatto81@gmail.com; 11 Dipartimento Cardiotoracico, Ospedale “Santa Maria della Misericordia”, Azienda Sanitaria Universitaria Friuli Centrale (ASUFC), 33100 Udine, Italy; luca.franchin@gmail.com; 12 Cardiologia Levante, P.O. Levante-Ospedale San Paolo, 17100 Savona, Italy; stefano.cornara@gmail.com; 13 U.O.C. Cardiologia-UTIC, Ospedale “Monsignor R. Dimiccoli”, 76121 Barletta, Italy; michele.magnesa17@gmail.com; 14 U.O.C. Cardiologia, Azienda Ospedaliero-Universitaria Senese, 53100 Siena, Italy; carlotta.sorinidini@gmail.com (C.S.D.); enri91@gmail.com (E.V.); 15 Division of Cardiology, AULSS2 Marca Trevigiana, Ca’ Foncello Hospital, 31100 Treviso, Italy; gasparetto.nicola@gmail.com; 16 Division of Cardiology, Emergency Department and Critical Areas, Azienda Ospedaliera Santa Croce e Carle, 12100 Cuneo, Italy; roberta.rossini2@gmail.com; 17 Cardiovascular Disease Unit, IRCCS Ospedale Policlinico San Martino, 16132 Genoa, Italy; roberta.dellabona@gmail.com; 18 Cardiology Unit, Santo Spirito Hospital, Casale Monferrato, 15033 Alessandria, Italy; federico.nardi1@gmail.com; 19 U.O.C. Cardiologia-UTIC, Azienda Ospedaliera San Camillo Forlanini, 00152 Roma, Italy; dgabrielli@scamilloforlanini.rm.it; 20 “A. De Gasperis” Cardiovascular Department, Division of Cardiology, ASST Grande Ospedale Metropolitano Niguarda, Piazza dell’Ospedale Maggiore 3, 20162 Milan, Italy; fabri.oliva@gmail.com; 21 Cardiovascular and Thoracic Department, University Cardiology, AOU Città della Salute e della Scienza di Torino, 10126 Torino, Italy; massimo.imazio@uniud.it

**Keywords:** colchicine, inflammation, residual cardiovascular risk, coronary artery disease

## Abstract

Atherosclerotic coronary artery disease remains a leading cause of morbidity and mortality worldwide, despite advances in lipid-lowering and antithrombotic therapies. Increasing evidence highlights the pivotal role of inflammation in all stages of atherosclerosis, from plaque formation to rupture. Colchicine, a well-known anti-inflammatory drug traditionally used in gout and pericarditis, has emerged as a promising agent in the secondary prevention of cardiovascular events. Recent clinical trials have demonstrated significant reductions in cardiovascular outcomes with low-dose colchicine, especially in patients with stable CAD and following myocardial infarction. This review provides an updated overview of the pathophysiological rationale for colchicine use in atherosclerosis, summarizes key clinical trial data, and discusses potential mechanisms, safety considerations, and future directions.

## 1. Introduction

Coronary artery disease (CAD) is the leading cause of death worldwide [1]. Several risk factors increase the risk of developing CAD; these can be classified into non-modifiable (genetics, age, sex) and modifiable (lipid, smoke, arterial hypertension) [2]. The “lipid hypothesis” is usually considered the main pathological mechanism of atherosclerosis; according to it, lipid accumulation into the intima layer of the artery is the trigger of this disease, and the risk increases proportionally with blood lipid concentration [3]. But recently this hypothesis has been updated because it has been demonstrated that oxidated LDL (Low-density lipoprotein) cholesterol and macrophages pathologically accumulate into intima and start atherosclerosis [4,5]. These macrophages through the formation of NLRP3 (Nod-like receptor protein 3) inflammasome produce inflammatory cytokines (IL-1, IL-6, IL-18) [4]. A modern view considered atherosclerosis as a progressive inflammatory process triggered by coronary risk factors. Indeed, chronic inflammatory auto-immune diseases (rheumatoid arthritis, systemic lupus erythematosus) and human immunodeficiency virus (HIV) infection are considered risk factors for CAD that confer high risk [6,7]. In patients treated with lipid-lowering drugs, low-grade systemic inflammation determined by high sensitivity C-reactive protein (hsCRP) is the most powerful determinant of recurrent cardiovascular events, cardiovascular death, and all-cause mortality [8]. This has been defined as residual inflammatory risk [8]. Considering the importance of inflammation in the development of atherosclerosis, numerous trials have sought to demonstrate a reduction in cardiovascular events with anti-inflammatory therapy in patients with established coronary artery disease (methotrexate [9], canakinumab [10], colchicine [11]) but all have failed except for canakinumab and colchicine [11]. Colchicine is an alkaloid originally extracted from plants of the genus Colchicum (in particular, Colchicum autumnale) [12]. Colchicine has been used since ancient times as an anti-inflammatory to treat gout attacks and in elevated dose as poison [12]. The aim of this manuscript is to review the role of colchicine in current atherosclerotic cardiovascular disease (ASCVD) management.

## 2. Mechanism of Action of Colchicine in Atherosclerosis

Colchicine is an established anti-inflammatory drug currently indicated mainly for the treatment of inflammatory conditions such as pericarditis [13], gout [14], and familial Mediterranean fever (FMF) [15]. Due to its potent anti-inflammatory and immunomodulatory properties, it is also considered a promising candidate for combating atherosclerosis. Chemically, colchicine is a small lipophilic molecule composed of three rings (A, B, and C). Rings A (trimethoxyphenyl moiety) and C (methoxytropone moiety) primarily mediate its binding to tubulin, while ring B maintains the structural rigidity required for its biological activity [16]. Its main mechanism of action involves effects inhibiting tubulin polymerization, which disrupts the cellular cytoskeleton and impairs essential cellular functions, such as mitosis, intracellular transport, and phagocytosis [17]. This drug exerts a concentration-dependent biphasic effect on microtubules: at lower concentrations, it inhibits microtubule growth, whereas at higher concentrations, it induces their depolymerization. Such cytoskeletal disruptions compromise various cellular activities, critical for numerous physiological processes in healthy cells [18].

**Figure 1:**
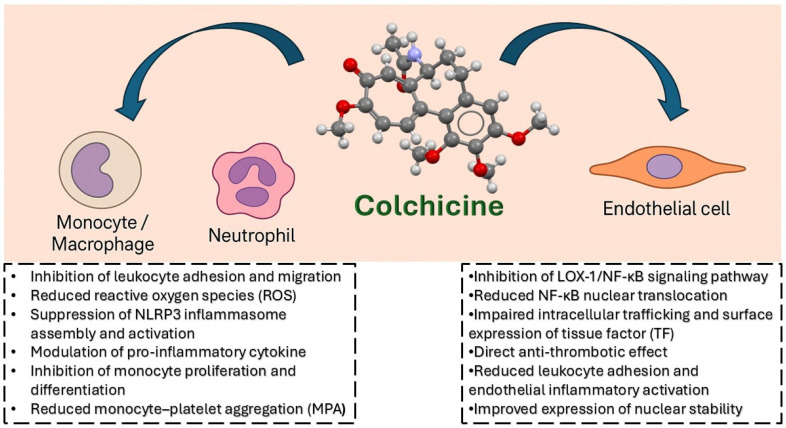
Mechanisms of action of colchicine on innate immune and endothelial cells in the context of atherosclerosis: Colchicine exerts pleiotropic anti-inflammatory and antithrombotic effects by modulating multiple cellular pathways. In innate immune cells, including monocytes, macrophages, and neutrophils, colchicine reduces leukocyte adhesion and migration, inhibits reactive oxygen species (ROS) production, suppresses NLRP3 inflammasome assembly and activation, modulates cytokine release, inhibits monocyte proliferation and differentiation, and reduces monocyte–platelet aggregation (MPA). In endothelial cells, colchicine interferes with the LOX-1/NF-κB signaling pathway, reduces NF-κB nuclear translocation, impairs microtubule-dependent trafficking and surface expression of tissue factor (TF), and exerts direct anti-thrombotic effects. It also attenuates endothelial inflammatory activation and improves nuclear stability by modulating the expression of key DNA repair proteins. These mechanisms collectively contribute to the therapeutic potential of colchicine in atherosclerosis.

Overall, the biological actions of colchicine on neutrophils, monocytes, the NLRP3 inflammasome, cytokines, and endothelial function provide a strong mechanistic basis for its potential role in preventing or slowing the progression of atherosclerosis (Figure 1).

### 2.1. Effects on Neutrophils and Monocytes

Colchicine interferes with neutrophil activity, a contributor to vascular inflammation and plaque destabilization. At nanomolar concentrations, colchicine disrupts E-selectin distribution on endothelial cells, reducing neutrophil recruitment and transmigration at inflammatory sites. [19]. At higher micromolar concentrations, it promotes L-selectin shedding from neutrophils, further hindering their recruitment into inflamed tissues [20].

Additionally, colchicine affects neutrophil deformability and motility by altering cellular viscoelastic properties. Specifically, colchicine reduces cytoplasmic elasticity through partial microtubule depolymerization, counterbalanced by increased elasticity and viscosity in the plasma membrane compartment due to enhanced subcortical actin filament formation. These cytoskeletal changes impair neutrophil deformability, thus restricting their ability to migrate through confined intercellular spaces, a crucial step in inflammation-related extravasation [21].

Colchicine also inhibits superoxide production in neutrophils triggered by monosodium urate (MSU) crystals, an important source of oxidative stress, by blocking early signaling events, such as tyrosine phosphorylation [22].

Monocytes also play a pivotal role in the pathogenesis of atherosclerosis [23]. Colchicine exerts significant inhibitory effects on monocyte-driven inflammation; in particular, experimental in vitro evidence highlights that colchicine directly modulates monocyte functions, reducing their proliferation rates and impeding their differentiation, critical steps in the pathogenesis and progression of atherosclerotic plaques [24]. Furthermore, colchicine also inhibits monocyte–platelet aggregation (MPA), and lowers the expression of platelet activation markers like P-selectin, thus dampening vascular inflammation and thrombotic risk [25].

### 2.2. Inhibition of the NLRP3 Inflammasome

The NLRP3 inflammasome, mainly active in macrophages, is a key regulator of innate immunity by controlling the maturation and release of pro-inflammatory cytokines, most notably interleukin-1β (IL-1β) [26]. Within the context of atherosclerosis, cholesterol crystals (CCs) represent a major endogenous activator of the NLRP3 inflammasome, inducing a robust inflammatory response [27]. However, it can also be activated by additional stimuli, including MSU and extracellular adenosine triphosphate (ATP) [28]. Several experimental findings have consistently demonstrated that colchicine exerts a potent inhibitory effect on NLRP3 inflammasome activation. Initial studies provided evidence of effective NLRP3 inhibition by colchicine using in vitro cell-based models; specifically, colchicine was shown to reduce inflammasome activity in THP1 cells stimulated with MSU crystals [29]. Subsequent clinical studies further supported this observation, demonstrating a marked suppression of IL-1β secretion by colchicine in patients with FMF [30]. Additional experimental data revealed a clear dose-dependent inhibition of IL-1β production by colchicine in macrophages exposed to several inflammasome-activating stimuli, including MSU crystals and extracellular ATP [31]. Colchicine is thought to hinder the proper intracellular movement of ASC (apoptosis-associated speck-like protein containing a C-terminal caspase recruitment domain), a critical adaptor protein, thereby impairing the spatial organization required for NLRP3 inflammasome activation [31]. Further mechanistic insights into colchicine’s anti-inflammasome activity have emerged, emphasizing additional targets at a molecular level. For instance, colchicine significantly reduces the levels of activated caspase-1 and mature IL-1β proteins, without substantially influencing the transcription of NLRP3 or IL-1β genes, indicating a selective post-translational mechanism of action [32]. Additionally, it can directly bind the NLRP3 protein’s ATP-binding site and inhibit the ATP-dependent P2 × 7 receptor pore formation, further preventing inflammasome activation [33,34]. These actions highlight colchicine’s broad, multi-target inhibition of NLRP3 inflammasome-driven inflammation, supporting its therapeutic potential in inflammatory-driven diseases and atherosclerosis [35,36].

### 2.3. Modulation of Cytokines and Endothelial Function

Colchicine exerts protective effects on endothelial cells primarily by targeting critical inflammatory pathways involved in endothelial dysfunction. In patients with FMF, it significantly reduces circulating endothelial markers like asymmetric dimethylarginine (ADMA), thrombomodulin (TM), and osteoprotegerin (OPG), clearly reflecting its endothelial protective properties [37].

A central driver of endothelial damage in atherosclerosis is oxidative stress response from oxidized low-density lipoproteins (oxLDLs) [38] OxLDL interacts specifically with the lectin-like oxidized LDL receptor-1 (LOX-1), thereby triggering several pro-inflammatory signaling cascades, particularly involving activation of the nuclear transcription factor NF-κB [39]. This transcription factor can also be activated by various other environmental insults, including genetic mutations, infections, pathogenic autoantibodies, proinflammatory cytokines, and cigarette smoking [40]. Once activated, NF-κB enhances the release of pro-inflammatory cytokines, including IL-6, IL-8, and TNF-α, which further amplify endothelial inflammation by recruiting neutrophils and monocytes [41].

OxLDL also increases expression of tissue factor (TF), a critical mediator of coagulation and thrombosis, by promoting its transport to the cell surface [42,43].

Colchicine interferes with TF transport by binding to β-tubulin, reducing its surface expression and lowering thrombotic risk. [44]. It also blocks NF-κB nuclear translocation and consequently reduces the expression of TF and other pro-inflammatory mediators triggered by oxLDL [45].

Additionally, colchicine protects against oxidative stress-induced endothelial aging by attenuating the H_2_O_2_-induced upregulation of p21 expression, preserving nuclear integrity through the prevention of KU80 and Lamin B1 downregulation, and inhibiting the phosphorylation of key signaling molecules involved in stress responses, including p38, ERK, AKT, and mTOR [46].

## 3. Clinical Evidence in Acute Coronary Syndromes

Acute coronary syndromes (ACSs) remain a major global health burden. Despite optimal medical therapy and timely revascularization, many patients continue to face a high residual risk of recurrent ischemic events. Increasing evidence underscores the pivotal role of inflammation in the pathophysiology of atherosclerosis and its complications. The landmark Canakinumab Anti-inflammatory Thrombosis Outcomes Study (CANTOS) was the first large randomized trial to demonstrate that targeting inflammation can reduce cardiovascular events independently of lipid lowering [10]. Building on these findings, the COLCOT (Colchicine Cardiovascular Outcomes Trial) trial investigated low-dose colchicine—an affordable, well-established, and widely available anti-inflammatory agent—in the post-ACS setting [47]. The COLCOT was a randomized, double-blind, placebo-controlled trial enrolling 4745 patients who had experienced an MI within 30 days (median time to randomization: 13.5 days). Participants received colchicine 0.5 mg once daily or placebo, in addition to guideline-directed medical therapy [47]. The primary endpoint was a composite of cardiovascular death, resuscitated cardiac arrest, myocardial infarction, stroke, and urgent hospitalization for angina leading to coronary revascularization. The authors found that colchicine significantly reduced the primary endpoint by 23% (HR 0.77; 95% CI, 0.61–0.96; *p* = 0.02) [47]. Notable reductions were observed in stroke (HR 0.26; 95% CI, 0.10–0.70; *p* = 0.007) and urgent hospitalization for angina (HR 0.50; 95% CI, 0.31–0.81; *p* = 0.006). Nevertheless, adverse events were slightly more frequent in the colchicine group—particularly gastrointestinal symptoms and pneumonia—but were generally manageable and did not outweigh the cardiovascular benefits observed [47]. The positive findings of COLCOT have led to guideline updates. The ESC 2023 guidelines now recommend considering low-dose colchicine for secondary prevention in patients with recent MI and no contraindications (Class IIb recommendation) [48]. However, caution is warranted as colchicine is metabolized via CYP3A4 and P-glycoprotein and care is needed in polypharmacy settings. Moreover, higher rates of gastrointestinal intolerance and rare but notable risks of infection (e.g., pneumonia) have been reported. Ultimately, colchicine offers a novel, cost-effective adjunct for patients with persistent inflammation after ACS, particularly when traditional risk factors are well controlled but residual inflammatory risk remains.

The onset of acute myocardial ischemia triggers an initial pro-inflammatory response in order to remove necrotic cell debris from the infarct area and initiate tissue repair. However, the reperfusion secondary to primary percutaneous intervention can exacerbate this pro-inflammatory response, contributing to additional cardiomyocyte death and myocardial tissue injury—a phenomenon known as ‘myocardial reperfusion injury’, which manifests within 6 to 24 h after reperfusion [49]. This initial local inflammatory phase extends into a systemic response, as evidenced by a rise in circulating pro-inflammatory markers—most notably hs-CRP, which peaks 2–3 days after myocardial infarction, and interleukin-6 (IL-6). The biological rationale for colchicine in ACS stems from its action on the NLRP3 inflammasome, a key component in the inflammatory response driving atherogenesis and plaque instability. Colchicine disrupts microtubule polymerization, reducing neutrophil chemotaxis and IL-1β/IL-6 production [50]. These cytokines are implicated in post-MI inflammation, adverse remodeling, and recurrent plaque rupture. Importantly, in the COLCOT trial, colchicine was initiated within 30 days of MI, with subgroup analysis suggesting greater benefit when therapy began within 3 days [47]. This early intervention supports a therapeutic window where inflammation is particularly active and modifiable.

Several sub-analyses of the COLCOT trial have been conducted. In the pre-specified subgroup of patients with type 2 diabetes and recent myocardial infarction [51], colchicine was associated with a 35% relative risk reduction in the primary efficacy endpoint compared with placebo. An interesting sub-analysis [8] showed that when colchicine was initiated within the first 3 days post-MI, the primary endpoint occurred in 4.3% of patients in the colchicine group versus 8.3% in the placebo group (*p* = 0.007). When treatment was started between Days 4 and 7 (*n* = 720), the event rates were 6.0% and 5.9%, respectively, and when initiated on Day 8 or later (*n* = 2748), the rates were 5.7% and 7.1%, respectively [52]. However, these differences were not statistically significant, suggesting, as previously mentioned, that early initiation (within 3 days) may confer greater benefit.

Further evidence on colchicine use in clinical practice comes from the recent CLEAR SYNERGY (OASIS 9) randomized trial [53], which investigated its efficacy in patients with acute myocardial infarction undergoing PCI. Conducted during the COVID-19 pandemic, the trial did not demonstrate a significant benefit of colchicine over placebo at a median follow-up of 3 years, providing inconsistent data regarding its efficacy. Notably, CLEAR SYNERGY is the largest clinical trial on colchicine in the acute coronary syndrome setting, with the highest number of recorded events (649 vs. approximately 300 in COLCOT). Nevertheless, it has significant limitations to be acknowledged. Conducted during the COVID-19 pandemic, it faced several limitations. The colchicine dosing protocol was altered during the study from 0.5 mg twice daily to 0.5 mg once daily, to reduce gastrointestinal side effects, but high doses were not used in previous trials, such as the LoDoCo trials [11,12] and COLCOT trial for acute coronary syndromes [13]. Outcome reporting may have suffered during the pandemic, leading to potential underreporting of MACEs. Adherence was based on self-reporting, which is inherently unreliable. Additionally, the possible inclusion of hemorrhagic events in stroke events adds potential confusion.

A recent meta-analysis [54] including 21,800 patients (colchicine *n* = 10,871; placebo *n* = 10,929) with follow-up ranging from 12 to 34 months demonstrated a 25% reduction in the incidence of major adverse cardiovascular events (MACEs) with colchicine compared to placebo 53. While the CLEAR SYNERGY trial alone did not show significant benefits, pooled data from randomized controlled trials provide a higher level of evidence, supporting the use of long-term colchicine therapy in patients with vascular disease, in line with current guideline recommendations—although these were issued prior to the publication of the most recent trials. Further evidence in this interesting field is needed.

## 4. Clinical Evidence in Chronic Coronary Syndromes

The role of colchicine in patients suffering with chronic coronary syndrome (CCS) has been validated in two randomized controlled trials (RCTs): the Low-Dose Colchicine for Secondary Prevention of Cardiovascular Disease (LoDoCo) and LoDoCo2 trials [55,56].

The LoDoCo trial was a prospective, open-label, randomized study which was designed for assessing the impact of colchicine 0.5 mg/day on the occurrence of the composite endpoint: acute coronary syndrome (ACS), fatal or nonfatal out-of-hospital cardiac arrest, or non-cardioembolic ischemic stroke [55]. Specifically, 532 patients were randomized to colchicine 0.5 mg/day or no colchicine and follow-up for 3 years. Patients were on optimal medical therapy as 93% of them were on aspirin and/or clopidogrel and 95% were on statins.

Colchicine was able to reduce the occurrence of the primary endpoint by 67% (Hazard Ratio [HR] 0.33, 95% confidence interval [CI] 0.18–0.59; *p* < 0.001) while demonstrating an outstanding number-needed-to-treat (NNT) equal to 11. The result was mainly driven by the reduction in relative risk of ACS (HR 0.33, 95% CI 0.18–0.63; *p* < 0.001), while there was no impact on cardiac arrest and non-cardioembolic stroke when each surrogate endpoint was considered. Stent and non-stent-related ACS were effectively reduced when colchicine was added to this specific population [55]. Although the study’s open-label design and relatively small sample size might be considered as possible limitations, the magnitude of benefit observed raised considerable interest in colchicine as a secondary prevention strategy in CCS.

The LoDoCo trial paved the way for the LoDoCo2 trial [56]. This was a randomized, controlled, double-blind trial. It enrolled 5522 patients with CCS who were randomized to receive 0.5 mg of colchicine once daily or placebo. The primary endpoint was a composite of cardiovascular death, spontaneous (nonprocedural) myocardial infarction, ischemic stroke, or ischemia-driven coronary revascularization [56]. Colchicine 0.5 mg once daily significantly reduced the occurrence of the primary endpoint (HR 0.69; 95% CI, 0.57 to 0.83; *p* < 0.001).

Specifically, these results seemed to be triggered by the higher volume and burden of calcified plaque, as well as the higher volume of dense calcified plaque compared with placebo and independently from the use of statins [57]. No influence of colchicine on pericoronary inflammation was demonstrated [57]. A LoDoCo2 biomarker substudy [58] rather found a reduction in plasma concentrations of extracellular vesicle (EV) NLR family pyrin domain containing 3 (NLRP3) inflammasome. As NLRP3 is already known to promote vascular inflammation and atherosclerosis development [59], its inhibition from colchicine might explain one of the possible mechanisms involved in cardiac protection from this compound in patients with CCS.

The plaque stabilization and the reduction of their inflammatory background is more evident when the treatment is prolonged. Insights from the LoDoCo2 trial [60] outlined the great divergence of the ROC curves in prolonged follow-up: from a relative risk reduction at the end of 1 yr follow-up of 32%, the 4 yr follow-up showed a relative risk reduction of 47%. Therefore, the long-term benefit of colchicine is plausible in patients with CCS.

Interesting aggregate data from the Low-Dose Colchicine 2 (LoDoCo2) trial and the Utrecht Cardiovascular Cohort-Second Manifestations of ARTerial disease (UCC-SMART) study [61] demonstrated a 4.6% reduction in median 10 yr absolute risk reduction (ARR) for MACEs and 8.6% reduction in MACEs plus coronary revascularization. When translated into lifetime benefit, there were 2 MACE-free years and 3.4 MACE plus coronary revascularization-free life-years gained.

Despite these confirmatory results on a wide sample size, the LoDoCo2 trial demonstrated higher incidence in death from non-cardiovascular causes in the colchicine group than in the placebo group (HR 1.51; 95% CI, 0.99 to 2.31) [56]. Such a difference was not statistically significant but might be detrimental for the successful implementation of the drug in the clinical daily setting. Mild gastrointestinal side effects, such as diarrhea and nausea, were more frequent in the colchicine group (9.7% vs. 8.2%), though these rarely led to treatment discontinuation [56].

Although no significant increase in infection risk or myotoxicity was observed, attention should be paid when colchicine is co-administered with certain statins (especially simvastatin or atorvastatin at higher doses), due to potential drug–drug interactions involving cytochrome P450 metabolism and P-glycoprotein transport (see Section 5 for details).

The main characteristics of the major clinical trials and meta-analyses evaluating the efficacy of low-dose colchicine in patients with CAD are shown in Table 1.

## 5. Safety Profile and Drug Interactions

Colchicine exhibits dose-dependent effects, and most side effects resolve with dose reduction or drug discontinuation. Its elimination occurs primarily via hepatic metabolism, predominantly through the CYP3A4 isoform of cytochrome P450. This metabolic pathway involves the demethylation of colchicine into less active metabolites, followed by biliary excretion, which accounts for approximately 60–80% of its clearance. P-glycoprotein (P-gp), an ATP-dependent efflux transporter located in enterocytes, hepatocytes, renal cells, and the blood–brain barrier, also contributes to colchicine metabolism. In enterocytes, P-gp actively expels colchicine back into the intestinal lumen, thereby limiting its systemic absorption. Renal excretion contributes to 20–40% of colchicine clearance. Notably, impaired hepatic or renal function significantly reduces colchicine clearance, increasing the risk of toxicity [62,63].

### 5.1. Gastrointestinal Side Effects and Dose Consideration

It is well documented that patients taking therapeutic oral doses of colchicine may experience a range of gastrointestinal symptoms, including abdominal pain, cramping, hyperperistalsis, diarrhea, nausea, and vomiting, occurring in 5–10% of cases [63,64]. These adverse effects are dose-dependent and can often be mitigated by dose reduction.

Gastrointestinal and coagulation disturbances have been reported following ingestion of doses less than 0.5 mg/kg. More serious toxic effects, including bone marrow aplasia and a mortality rate of up to 10%, have been observed with ingestions of 0.5–0.8 mg/kg [63]. Acute ingestions exceeding 0.8 mg/kg are frequently fatal.

The risk of colchicine toxicity is generally dose-dependent. However, the drug has a narrow therapeutic index, with significant overlap between therapeutic and toxic doses [63,64]. Doses of 0.5–0.8 mg/kg are considered highly toxic, while doses above 0.8 mg/kg are typically lethal.

It is important to note that extracorporeal elimination techniques (e.g., hemodialysis) are largely ineffective due to colchicine’s large volume distribution [63]. To reduce the risk of irreversible bolus overdose, the FDA has withdrawn approval for the intravenous formulation of colchicine [63].

### 5.2. Risks in Patients with Hepatic or Renal Impairment

Patients with impaired renal or hepatic function are at increased risk of colchicine toxicity and require close monitoring, even when receiving standard doses. Long-term use of colchicine at doses up to 1 mg daily is generally considered safe in patients without advanced kidney or liver disease, even when co-administered with other medications, and typically does not require dose adjustments.

In patients with mild renal or hepatic impairment, a daily dose of 1 mg has been used; however, it is advisable to reduce the dose to 0.5 mg per day due to the elevated risk of serious drug–drug interactions, particularly with medications that inhibit CYP3A4 or P-gp.

For patients with severe renal or hepatic impairment in whom colchicine therapy is essential, the dose should be limited to 0.5 mg no more than every other day to minimize the risk of accumulation and toxicity.

### 5.3. Interaction with Concomitant Medications

Table 2 describes the interaction between colchicine and several commonly used drugs and illustrate some strategies to prevent side effects arising from their concomitant use. As can be seen, most of these interactions arise from the interference with colchicine clearance through CYP3A4 and/or P-gp inhibition.

Interactions between colchicine and drugs or compounds that inhibit CYP3A4 can significantly increase serum and tissue concentrations of colchicine, leading to toxicity. Notable CYP3A4 inhibitors known to interact with colchicine include clarithromycin, erythromycin, ketoconazole, and natural grapefruit juice; however, numerous other inhibitors may also contribute to clinically relevant interactions [65,66,67,68].

The concomitant use of colchicine with macrolide antibiotics, particularly clarithromycin, has been associated with a markedly increased risk of hepatic failure and mortality [69]. In addition, P-glycoprotein (P-gp) inhibitors—such as clarithromycin and ciclosporin—also interfere with colchicine elimination and may enhance its toxicity.

The concomitant use of colchicine with strong CYP3A4 or P-gp inhibitors is contraindicated in patients with renal or hepatic impairment. Even in patients with normal hepatic and renal function, a dose reduction or temporary discontinuation of colchicine should be considered when these inhibitors are prescribed.

Colchicine and statins are frequently co-administered for the treatment and prevention of cardiovascular conditions, auto-inflammatory diseases, and gout. While this combination may offer clinical benefit, it is associated with an increased risk of myotoxicity, ranging from mild myalgia to severe rhabdomyolysis. The interaction between colchicine and statins is complex and not yet fully understood.

Although the exact incidence of colchicine-induced myotoxicity remains unclear, statin-associated myopathy occurs in 5–10% of cases, with rhabdomyolysis reported in approximately 0.01–0.1%. One proposed mechanism involves competition between colchicine and statins for CYP3A4 metabolism and P-gp-mediated elimination. Lipophilic statins (e.g., atorvastatin and simvastatin), which rely more heavily on CYP3A4 and P-gp pathways, appear to pose a greater risk for muscle toxicity compared to hydrophilic statins (e.g., rosuvastatin and pravastatin), which are less dependent on these routes [70,71].

## 6. Limitations and Controversies

Colchicine is an attractive candidate for modulating the inflammatory response that underlies atherosclerosis and its complications. However, despite these promising results, the clinical benefit of colchicine shows a degree of variability, highlighting the need to better understand optimal timing, duration, and patient selection to maximize therapeutic benefit while minimizing risk.

Variability in clinical benefit has been observed across studies and patient subgroups, suggesting that the anti-inflammatory effects of colchicine may not be universally beneficial or equally potent across all cardiovascular patients. For instance, while both COLCOT [47] and LoDoCo2 [56] demonstrated a significant reduction in composite cardiovascular endpoints, other studies have not replicated these benefits with the same magnitude, and some have noted an increased incidence of adverse gastrointestinal events or infections [72]. This heterogeneity may be attributable to differences in baseline inflammatory burden, genetic polymorphisms affecting colchicine metabolism, or comorbidities that influence drug response [73]. Moreover, the interaction between colchicine and other pharmacotherapies, such as statins or antiplatelet agents, could modify its efficacy or toxicity profile [74]. These findings underscore the necessity of identifying specific biomarkers or clinical characteristics that can predict colchicine responsiveness, enabling a more tailored approach to its use in cardiovascular disease management.

The question of optimal timing for colchicine initiation is also crucial. The COLCOT trial, which administered colchicine within 30 days of myocardial infarction, demonstrated a significant reduction in cardiovascular events, suggesting that early intervention may be more effective in curbing inflammatory cascades before irreversible damage occurs. Similarly, in CCS, where inflammation is more stable but persistent, long-term use of colchicine may help suppress low-grade inflammation and prevent future events. However, more data are needed to delineate the most effective duration of therapy. While short-term treatment may be insufficient to sustain anti-inflammatory benefits, indefinite treatment raises concerns about long-term safety, patient adherence, and drug interactions. Regarding long-term safety, despite concerns raised by the non-significant increase in non-cardiovascular mortality not related to infections or malignancies observed in the LoDoCo2 study, reassurances about the long-term safety of low-dose colchicine come from subsequent meta-analyses and over 50 years of clinical experience in patients with FMF [75,76,77,78]. Nonetheless, continued pharmacovigilance is advisable.

Patient selection remains a central challenge in the use of colchicine for cardiovascular prevention. While the inclusion criteria in major trials were relatively broad, post hoc analyses suggest that certain subpopulations may derive greater benefit. For example, patients with elevated inflammatory markers such as hs-CRP may experience greater risk reduction with colchicine therapy [74], consistent with the notion of targeting residual inflammatory risk. Additionally, patients with metabolic syndrome, diabetes, or previous revascularization may constitute high-risk groups that may benefit from the drug. Conversely, individuals with a history of gastrointestinal intolerance, hepatic or renal impairment, or potential drug–drug interactions—especially involving CYP3A4 inhibitors or P-glycoprotein substrates—may be poor candidates for colchicine therapy [74]. The development of clinical algorithms or scoring systems to guide colchicine prescribing in cardiovascular disease could enhance therapeutic precision and minimize unnecessary exposure in low-yield patients.

These considerations highlight the broader imperative for personalized anti-inflammatory strategies in patients with CAD. Colchicine’s modest cost and oral formulation make it accessible, but its utility will be maximized when deployed as part of a comprehensive strategy that takes into account the inflammatory profile, comorbid conditions, and risk of adverse effects. Emerging research into inflammation-specific imaging modalities may eventually enable cardiologists to stratify patients more precisely and select the most appropriate anti-inflammatory agents based on biological rather than purely clinical criteria [79]. Such precision medicine approaches could substantially improve outcomes while minimizing the risk of overtreatment.

From a health economics standpoint, colchicine appears to be a cost-effective intervention for secondary prevention of cardiovascular events. Its low cost, combined with its ability to reduce the incidence of expensive-to-treat events such as myocardial infarction, stroke, and coronary revascularization, results in favorable cost-effectiveness ratios in most analyses [80]. However, the cost-effectiveness of colchicine is contingent upon patient adherence over the long term. Although colchicine is generally well tolerated, gastrointestinal side effects such as diarrhea and nausea are common and can lead to discontinuation [79]. Additionally, the need for chronic daily dosing in a population that is often already taking multiple medications for CV risk factors treatment can pose challenges for adherence. Continued research into lower or intermittent dosing regimens could potentially maintain efficacy while reducing adverse events and improving tolerability.

## 7. Practical Considerations for Clinical Use

According with the class IIa indication (level of evidence A) established in the Guidelines published by the European Society of Cardiology (ESC) in 2024, colchicine should be prescribed to adult patients with chronic coronary syndromes (CCSs) due to atherosclerotic coronary artery disease [81].

As regards Acute Coronary Syndromes (ACSs), in the Guidelines published by the European Society of Cardiology (ESC) in 2023 is stated that colchicine may be considered, particularly if other risk factors are insufficiently controlled or if recurrent cardiovascular disease events occur under optimal therapy (class IIb indication) [48]. For both indications, it has been suggested that an elevated hs-CRP could help in identifying patients with a more relevant inflammatory burden, that could therefore receive a greater clinical benefit from this treatment. However, evidence in this field is lacking, because large clinical trials were not restricted to this subset of patients [82]. Neither the LoDoCo trials or COLCOT selected patients based on elevated inflammatory risk, currently defined as hs-CRP >2 mg/L [47,55,56]. A subgroup of COLCOT showed higher median baseline CRP concentrations after myocardial infarction [47], but the lack of significant differences in reductions over time suggests that colchicine may benefit a wider range of CAD patients. A biomarker-specific strategy could help identify a targeted patient subset, improving treatment precision and the benefit/risk ratio.

The 2020 proteomic study supports this by demonstrating significant reductions in hs-CRP and proteins related to the NLRP3 inflammasome and neutrophil function [83]. However, the absence of correlation between some protein reductions and changes in hs-CRP suggests that our definition of high inflammatory risk may require refinement.

Insights may emerge from the ongoing CLEAR SYNERGY biomarker study whereas pharmacogenomic determinants of colchicine safety have already been identified in COLCOT [84]. Future studies will be essential for understanding the relationship between inflammatory markers and clinical outcomes, particularly in patients with pronounced inflammatory profiles. Additionally, imaging techniques like coronary CT angiography and transcriptomic profiles are being explored for patient selection [85].

Moreover, based on two small studies showing conflicting results on incidence of restenosis [86,87], and a single study showing that colchicine can prevent a rise in blood markers of vascular inflammation during an acute injury [88], colchicine may be considered in selected patients undergoing percutaneous revascularization, e.g., patients with previous stent restenosis despite guideline-directed medical therapy, patients with metabolic syndrome, or with high risk features such as multivessel disease, or treatment of lesions involving bifurcations.

A dose of 0.5 mg once daily is suggested, without adjustment for body weight, age, or renal or hepatic function, both in CCS and in ACS [48,81]. In this latter case, initiation of treatment within 3 days from presentation should be considered to obtain the greater clinical benefit [89].

**Figure 2:**
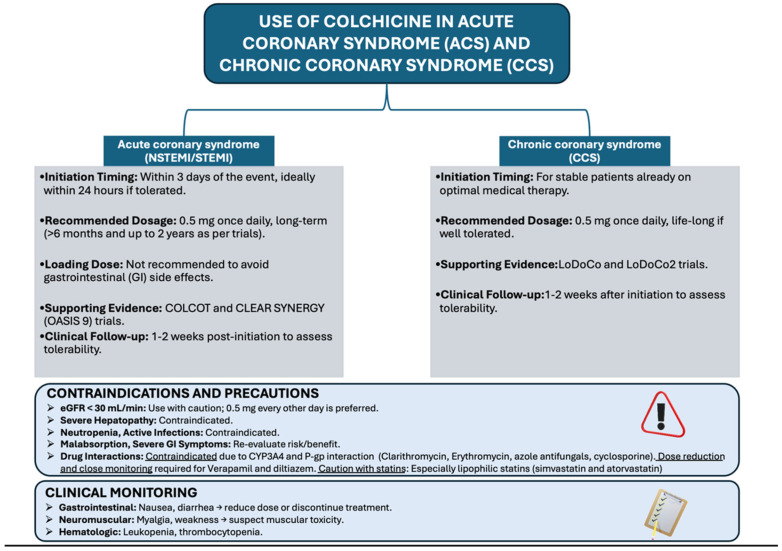
Algorithm for use of colchicine in acute coronary syndrome (ACS) and chronic coronary syndrome (CCS) in clinical practice.

In selected patients undergoing percutaneous revascularization, e.g., patients with previous stent restenosis despite guideline-directed medical therapy, initiation of colchicine the day before or immediately after the intervention should be considered. Although the dose of drug in small studies focused on this setting [87,90] differed, based on more recent data on CCS, a dose of 0.5 mg once daily seems to be reasonable in this clinical scenario. Duration of treatment with colchicine is not clearly defined. However, based on the beneficial effects at longer follow-up [91], a treatment duration of at least 12 months after an ACS, and long-term treatment in CCS should be considered, in particular in patients with elevated markers of inflammation (such as hs-CRP), in patients with recurrent adverse cardiovascular events despite guideline-directed medical therapy, and in patients with other high risk features, such as multivessel coronary artery disease or ASCVD involving more than one vascular territory (e.g., CAD associated with peripheral artery disease or carotid artery stenosis). Nevertheless, temporary or definitive discontinuation of colchicine should be considered in patients with infectious disease, or with severe renal or hepatic impairment (Figure 2).

### Monitoring and Follow-Up

Patients on treatment with colchicine should be clinically evaluated at least once yearly to confirm the favorable benefits/risks ratio and adherence to guideline-directed medical therapy. A closer follow-up should be considered in patients with renal or hepatic impairment, and in patients with previous history of recurrent infections or at risk for infectious disease.

Monitoring of blood count, renal and liver function, and hs-CRP is indicated at least twice yearly, or more often in case of mild/moderate renal or hepatic impairment or in frail patients.

## 8. Future Perspectives and Unmet Needs

Despite mounting evidence supporting colchicine in ASCVD, several important gaps remain. Future research should aim to identify biomarkers capable of predicting which patients derive the greatest benefit, thereby improving risk stratification and minimizing unnecessary exposure. Dedicated trials in underrepresented populations—including women, older adults, and patients with CKD—are particularly warranted.

With respect to sex, although no significant sex–treatment interaction was reported in pivotal RCTs or a recent meta-analysis [92], the statistical power to detect sex-specific effects was limited. Regarding age, the mean age in major trials was in the early-to-mid 60s, leaving very elderly patients underrepresented. Some evidence suggests that benefits may attenuate beyond 65 years [93], while polypharmacy increases the risk of toxicity, particularly in the presence of CYP3A4/P-gp inhibitors.

Patients with moderate-to-severe CKD were largely excluded from existing RCTs. Current regulatory labeling contraindicates colchicine in advanced renal failure and in combination with strong CYP3A4/P-gp inhibitors, underscoring the need for careful patient selection, close monitoring, and CKD-specific studies. The ongoing ColchiRen trial (NCT06998862) is designed to address this evidence gap by assessing efficacy and safety in patients with moderate CKD.

At present, the role of colchicine in primary prevention remains unestablished. A recent Cochrane review concluded that the evidence for primary prevention is of very low certainty [94], while ongoing trials such as COLCOT-T2D and NCT05175274 will be critical to clarify both efficacy and long-term safety in this context.

Long-term safety, particularly regarding infection risk and drug–drug interactions, also requires further elucidation in real-world settings. In addition, the interaction of colchicine with other therapies that exert anti-inflammatory and plaque-stabilizing effects in ACS, such as PCSK9 inhibitors and SGLT2 inhibitors [95,96], merits deeper investigation. Other unresolved questions include the optimal duration of therapy, cost-effectiveness across different healthcare systems, and strategies to improve adherence. Ultimately, the refinement of personalized anti-inflammatory therapy represents a promising frontier in cardiovascular prevention.

## 9. Conclusions

Colchicine has emerged as a valuable therapeutic option in the management of ASCVD, because of its anti-inflammatory and immunomodulatory properties. Its mechanisms of action target key inflammatory pathways involved in atherosclerosis, particularly through inhibition of neutrophil activation, NLRP3 inflammasome assembly, and modulation of endothelial dysfunction. Clinical trials such as COLCOT and LoDoCo2 have demonstrated that low-dose colchicine can significantly reduce MACEs in both acute and chronic coronary syndromes. These benefits are particularly evident when colchicine is initiated early after acute coronary events and maintained long-term in chronic disease settings. Despite these positive outcomes, colchicine use is associated with a modest increase in non-cardiovascular adverse events, most commonly gastrointestinal disturbances, and requires caution in patients with hepatic or renal impairment due to its metabolism via CYP3A4 and P-glycoprotein pathways. Drug interactions, particularly with statins and macrolides, must also be carefully managed.

Current clinical guidelines recommend colchicine as an adjunctive therapy for secondary prevention in selected high-risk ASCVD patients, particularly when residual inflammatory risk remains despite optimal treatment of traditional risk factors. Nonetheless, further research is necessary to better define ideal patient selection, treatment duration, and long-term safety. In this context, colchicine represents a low-cost, accessible, and effective strategy to complement current cardiovascular therapies, offering targeted modulation of inflammation, an established driver of atherosclerosis and its clinical complications.

## Figures and Tables

**Table 1:** Summary of major clinical trials and meta-analyses evaluating the efficacy of low-dose colchicine in patients with coronary artery disease (CAD).

Trial	Setting	No. of Patients	Follow-Up	Primary Endpoint	Result
COLCOT [47]	Post-MI (<30 days)	4745	22 months	CV death, MI, stroke, angina → revascularization	−23% (HR 0.77; *p* = 0.02)
LoDoCo [55]	Chronic CAD	532	3 years	ACS, fatal or nonfatal out-of-hospital cardiac arrest, non-cardioembolic ischemic stroke	−67% (HR 0.33, *p* < 0.001)
LoDoCo2 [56]	Chronic CAD	5522	28.6 months	CV death, MI, ischemic stroke	−31% (HR 0.69; *p* < 0.001)
CLEAR SYNERGY [53]	Acute MI with PCI	7000+	3 years	CV death, MI, stroke	No significant benefit
Meta-analysis [54]	RCTs (21,800 patients)	10,871 colchicine 10,929 placebo	12–34 months	Composite MACE	−25% MACE vs. placebo

Abbreviations: MI: myocardial infarction; CAD: coronary artery disease; CV: cardiovascular; PCI: percutaneous coronary intervention; RCTs: randomized clinical trials; MACEs: major acute cardiovascular events.

**Table 2:** Drug interactions with colchicine and strategies to prevent toxicity.

Drug Class/Interaction Type	Examples	Mechanism	Clinical Risk	Preventive Strategies
CYP3A4 Inhibitors	Clarithromycin, Erythromycin, Ketoconazole, Fluconazole, Diltiazem, Grapefruit juice, Nefazodone	Inhibit hepatic metabolism of colchicine	Increased colchicine levels Enhanced risk of myopathy, GI toxicity, multiorgan failure	Avoid combination in patients with renal/hepatic impairment; consider dose reduction or temporary discontinuation of colchicine in others
P-gp Inhibitors	Ciclosporin, Azithromycin, Carvedilol, Erythromycin, Lopinavir, Propafenone, Tacrolimus	Inhibit colchicine efflux Increased intracellular levels	Enhanced risk of colchicine accumulation and toxicity	Contraindicated in renal/hepatic impairment; monitor closely and reduce colchicine dose if co-use is necessary
Dual CYP3A4 and P-gp Inhibitors	Amiodarone, Verapamil, Clarithromycin, Cyclosporine, Dronedarone, Itraconazole, Ketoconazole, Ranolazine	Block both metabolism and excretion	High potential for severe colchicine toxicity, incl. rhabdomyolysis and bone marrow suppression	Avoid colchicine or use very low doses with close monitoring; avoid repeat dosing for ≥2 weeks in case of flares
Statins (Lipophilic)	Atorvastatin, Simvastatin, Lovastatin	Compete for CYP3A4 and P-gp; additive myotoxicity	Enhanced risk of myopathy, rhabdomyolysis	Prefer hydrophilic statins (e.g., rosuvastatin, pravastatin); monitor CK and muscle symptoms
Immunosuppressants	Tacrolimus, Cyclosporine	P-gp inhibition; impaired colchicine clearance	Enhanced risk of systemic colchicine toxicity	Avoid combination if possible; otherwise, use minimal colchicine dose and monitor closely
Antifungals (Azoles)	Itraconazole, Ketoconazole	Strong CYP3A4 inhibitors	Enhanced risk of colchicine overexposure	Avoid combination or significantly reduce colchicine dose
Calcium Channel Blockers	Verapamil, Diltiazem	CYP3A4 and P-gp inhibition	Enhanced colchicine toxicity	Use alternative agents or reduce colchicine dose; monitor closely
Antivirals	Ritonavir, Lopinavir	Strong CYP3A4/P-gp inhibition	Increased colchicine levels, especially in COVID-19 treatment	Use alternative anti-inflammatory agents; avoid colchicine unless no alternative exists

## Data Availability

Data sharing is not applicable to this article as no new data were created or analyzed in this study.
